# Gastroesophageal Reflux Disease 10 Years After Bariatric Surgery—Is It a Problem? A Multicenter Study (BARI-10-POL)

**DOI:** 10.3390/jcm14155405

**Published:** 2025-07-31

**Authors:** Natalia Dowgiałło-Gornowicz, Monika Proczko-Stepaniak, Anna Kloczkowska, Paweł Jaworski, Piotr Major

**Affiliations:** 1 Department of General, Minimally Invasive and Elderly Surgery, Collegium Medicum, University of Warmia and Mazury, 10-045 Olsztyn, Poland; 2 Department of General, Endocrine and Transplant Surgery, Medical University of Gdansk, 80-214 Gdańsk, Poland; monika.proczko-stepaniak@gumed.edu.pl (M.P.-S.); anna.kloczkowska@gumed.edu.pl (A.K.); 3 Department of General, Oncological and Digestive Tract Surgery, Centre of Postgraduate Medical Education, Orłowski Hospital, 00-416 Warsaw, Poland; pawel.jaworski@cmkp.edu.pl; 4 2nd Department of General Surgery, Jagiellonian University Medical College, 30-688 Cracow, Poland; piotr.major@uj.edu.pl

**Keywords:** metabolic bariatric surgery, GERD, gastroesophageal-reflux disease, reflux, long-term follow-up

## Abstract

**Background/Objectives**: Gastroesophageal reflux disease (GERD) seems to be a common complaint which persists or develops after metabolic bariatric surgery (MBS). Endoscopic evaluation is vital in both the preoperative and postoperative phases to ensure optimal patient outcomes. The aim of this study was to evaluate the prevalence of GERD after MBS in a 10-year follow-up and analyze the endoscopic outcomes. **Methods**: This retrospective, multicenter study included 368 patients who underwent single bariatric procedure. The data came from five bariatric centers in Poland, part of the BARI-10-POL project. Data on symptoms of GERD, endoscopic findings, demographics, and surgical outcomes were collected for a 10-year follow-up period. Surgical procedures included SG, Roux-en-Y gastric bypass (RYGB), and one anastomosis gastric bypass (OAGB). **Results**: Of the 305 patients without symptoms of GERD, 12.3% developed de novo GERD postoperatively. There was no statistical significance regarding the new-onset symptoms and the type of MBS (*p* = 0.074) and the presence of symptoms of GERD and the type of MBS (*p* = 0.208). However, SG was associated with a significantly lower likelihood of GERD remission after MBS (*p* = 0.005). Endoscopic evaluation showed abnormal findings in asymptomatic patients in both preoperative (35.8%) and postoperative (14.1%) examinations (*p* < 0.001). **Conclusions**: GERD may be a common issue after MBS. One-quarter of patients after MBS may experience symptoms of GERD, regardless of the type of MBS. SG appears to be associated with a higher risk of persistent symptoms of GERD and a lower likelihood of GERD remission after MBS. Asymptomatic patients both before and after MBS may have abnormal findings in gastroscopy.

## 1. Introduction

Obesity has emerged as one of the most serious public health challenges globally, affecting over 2 billion people, approximately 30% of the world population [1]. Metabolic bariatric surgery (MBS) is currently the most efficacious treatment for obesity and obesity-related diseases [2,3]. However, there is a growing need for robust long-term data, particularly regarding the durability of weight loss or remission of obesity-related diseases. There are systematic reviews that provide foundational evidence supporting the sustained benefits of bariatric procedures over a 10-year period [4,5].

Among obesity-related comorbidities, gastroesophageal reflux disease (GERD) is especially prevalent, with up to 73% of patients considered for MBS experiencing symptoms of GERD [6]. Endoscopic evaluation plays a crucial role in the preoperative assessment and postoperative follow-up of patients [6]. Identifying existing esophageal conditions, such as esophagitis or Barrett’s esophagus, is essential for surgical planning. Preoperative endoscopy can detect these conditions, which may influence the choice of bariatric procedure. Similarly, postoperative endoscopic surveillance is essential to monitor for complications, including recurrent or de novo GERD, esophagitis, or development of Barrett’s esophagus [7].

The relationship between MBS and GERD varies depending on the surgical procedure. While sleeve gastrectomy (SG) is effective for weight loss, it has been associated with a high incidence of postoperative GERD. Studies have reported that up to 50% of patients develop new-onset symptoms of GERD after SG [8,9]. According to a study by Rebecci et al., the shape of the gastric sleeve appears to be one of the main factors predicting the risk of postoperative GERD [10]. GERD after one anastomosis gastric bypass (OAGB) is also a recognized complication. Although OAGB is effective for weight loss and metabolic benefits, the bile reflux component can contribute to GERD-like symptoms [11]. Roux-en-Y gastric bypass (RYGB) is often considered the gold standard for patients with obesity and GERD. It has been shown to reduce symptoms of GERD in the long term, with a study reporting a 21.1% rate of treated GERD at a 10-year follow-up [12]. Ashrafi et al. emphasized the importance of tailoring MBS to the patient’s preoperative GERD status, highlighting that careful patient selection is essential for optimal outcomes [13]. They suggest that in patients with pre-existing GERD or hiatal hernia, RYGB should be prioritized, while those undergoing SG require long-term monitoring due to the risk of developing or worsening GERD [13].

Routine endoscopy is recommended to monitor complications like GERD, esophagitis, or the development of Barrett’s esophagus. The International Federation for the Surgery of Obesity and Metabolic Disorders (IFSO) suggests performing an upper GI-tract endoscopy one year after MBS and then every 2–3 years following SG to enable early detection of potential issues [14]. Given the high prevalence and complex course of GERD in the bariatric population, both before and after MBS, comprehensive endoscopic evaluation is essential for optimizing patient outcomes.

The aim of this study was to evaluate the prevalence of GERD after MBS in a 10-year follow-up. The secondary aims are to assess the impact of individual surgical procedures on the occurrence of symptoms of GERD and to analyze preoperative and postoperative endoscopic results.

## 2. Materials and Methods

It was a retrospective, multicenter study of patients who underwent laparoscopic MBS in Poland between 2008 and 2014. It is part of the Bariatric Ten Years Outcomes in Poland (BARI-10-POL) project. The inclusion criteria were meeting the eligibility criteria for MBS (body mass index BMI > 40 or BMI > 35 with obesity-related diseases), having at least 10 years of follow-up data. Patients with a history of a previous bariatric procedure or missing or inconsistent records were excluded. Data were collected from five centers, under the patronage of the Metabolic and Bariatric Surgery Chapter.

The database consisted of information about GERD before and after the MBS: the presence of GERD, the need for chronic use of PPIs, preoperative and postoperative esophago-gastro—duodenal endoscopy (EGD) and its findings (hiatal hernia, Los Angeles classification grade of esophagitis (LA-), Barrett esophagus) [15]. All participating centers adhered to standardized diagnostic criteria for reporting endoscopic findings. Esophagitis was classified according to the Los Angeles classification grade system, while Barrett’s esophagus was reported based on the Prague C & M criteria. The presence of GERD was defined as typical symptoms of GERD (heartburn, regurgitation, epigastric pain, or sleep disturbances because of reflux at least twice a week for more than four weeks) regardless of use of PPIs and endoscopic findings. Additionally, the database included demographic data: sex, age, preoperative body mass index (BMI), the presence of obesity-related diseases (type 2 diabetes, hypertension, obstructive sleep apnea), the type of MBS, complications, and weight loss outcomes expressed as the percentage of excess weight loss (%EWL) and percentage of total weight loss (%TWL) [16]. The follow-up data were collected from hospital records, which were routinely generated during both in-person visits and telephone consultations. All reported results correspond to the 10-year follow-up period.

### 2.1. Surgical Techniques and Perioperative Care

The surgical procedures performed included SG, RYGB, and OAGB. All surgeries were performed in accordance with standard guidelines [17]. The presence of GERD did not influence the choice of surgical procedure. During SG, a 36F bougie was used, with gastric resection initiated 4–6 cm from the pylorus. In RYGB, the biliopancreatic limb measured approximately 100 cm, while the alimentary limb was about 150 cm in length. For OAGB, the biliopancreatic limb extended approximately 200 cm from the ligament of Treitz. If a large hiatal hernia (>5 cm in width) with intrathoracic migration (ITM) of the stomach was identified intraoperatively, hiatal hernia repair was performed. Each center involved in the study followed standardized protocols for preoperative, intraoperative, and postoperative care.

### 2.2. Statistical Analysis

All data were analyzed using Statistica software 13.PL (StatSoft Inc., Tulsa, OK, USA). A descriptive statistical analysis was conducted. The normal distribution was checked using the Shapiro–Wilk test. Due to the lack of a normal distribution, continuous variables were presented as medians with interquartile ranges. The Mann–Whitney U test was applied for continuous variables to compare two groups of patients. Categorical variables were compared using the chi-square test or Fisher’s exact test when appropriate. Logistic regression analysis was used to assess factors potentially associated with the occurrence of postoperative GERD. Univariable logistic regression was initially performed for each variable. As none of the variables reached statistical significance in the univariable analysis, multivariable logistic regression was not conducted. *p*-values  ≤  0.05 were considered statistically significant.

### 2.3. Ethical Considerations

The data were anonymized. The study was conducted in accordance with the ethical standards of the 1964 Declaration of Helsinki and its subsequent amendments. The study was approved by The Bioethics Committee of the University of Warmia and Mazury in Olsztyn (10/2024).

## 3. Results

**Figure 1:**
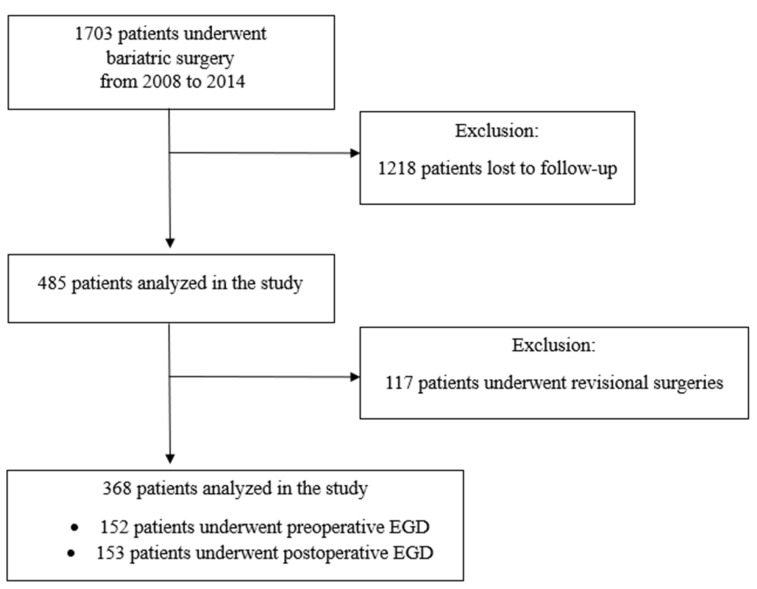
Flow chart of the study (EGD esophago-gastro-duodenoscopy).

The total population included 1703 patients. The patients who lost to follow-up were excluded from the study. In total, the BARI-10-POL database included 485 patients. The follow-up rate was 28.5%, Figure 1. For this analysis, we excluded patients with missing data and those who underwent revisional MBS. A total of 117 (24.1%) patients underwent revisional procedures, including 7 (6.0%) due to GERD and 13 (11.1%) due to GERD combined with concurrent weight regain, totaling 20 patients (17.2%). The analyzed group consisted of 368 patients (253 female, 68.8%).

In our analysis, 227 of 368 patients (61.7%) had no symptoms of GERD pre- or postoperatively.

### 3.1. Symptoms of GERD Before Surgery

A total of 63 of 368 patients (17.1%) reported symptoms of GERD before the MBS (Table 1). The patients with symptoms of GERD had significantly lower BMI preoperatively compared to patients without symptoms of GERD (*p* = 0.013). The most common MBS in both groups was SG (66.7%, 64.9%, respectively). There were no significant differences between the two groups in terms of age, type of MBS, presence of obesity-related diseases, or postoperative outcomes (Table 1). There was no statistically significant association between preoperative symptoms of GERD and the symptoms of GERD after MBS (*p* = 0.816). Only 20.6% of patients with preoperative GERD continued to require PPIs postoperatively.

### 3.2. Symptoms of GERD After Surgery

**Figure 2:**
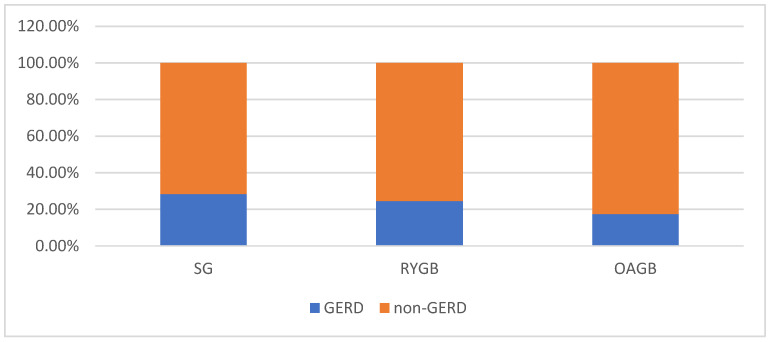
The prevalence of GERD after surgery depending on the type of procedure.

A total of 95 of 368 (25.8%) patients had symptoms of GERD after MBS, Table 2. The occurrence of symptoms of GERD was statistically significantly higher after the surgery than before (*p* = 0.004). No statistically significant differences were found between the groups with and without symptoms of GERD postoperatively in terms of characteristics or outcomes (Table 2). A total of 85.3% of those with symptoms of GERD reported the need for use of PPIs after the MBS. Among them, 57 underwent SG (23.8% of total of SG), 13 RYGB (20.0% of total of RYGB), and 11 OAGB (17.5% of total OAGB), (*p* = 0.513). Subjective symptoms of GERD after MBS were reported by 68 out of 240 patients who underwent SG (28.3%), 16 out of 65 after OAGB (24.6%), and 11 out of 63 after RYGB (17.5%) (*p* = 0.127), Figure 2.

All available factors potentially contributing to the prevalence of symptoms of GERD after MBS were analyzed using a univariable logistic regression model. No statistically significant associations were identified (Table 3). Multivariable logistic regression was not conducted.

### 3.3. EGD Findings

A total of 58.7% of patients did not undergo preoperative EGD (Table 4). Preoperatively, patients with symptoms of GERD were more likely to show pathological findings compared to those without symptoms of GERD (*p* < 0.001). A total of 46.5% of patients had hiatal hernia, 18.6% LA-A, and 4.7% LA-B. In contrast, only 64.2% of patients without symptoms of GERD had no changes (Table 4). A total of 58.4% of patients did not undergo the postoperative EGD. More patients with symptoms of GERD after MBS had abnormal postoperative EGD compared to patients without symptoms of GERD after MBS (*p* < 0.001). Among patients with symptoms of GERD after MBS, 29.4% had LA-B, and 11.8% at least LA-C. In contrast, 85.8% after MBS without GERD had no changes in postoperative EGD. There was no association between preoperative symptoms of GERD and postoperative changes in EGD (*p* = 0.683).

Of the patients with no preoperative endoscopic findings of GERD, 45 of 83 (54.2%) underwent postoperative gastroscopy. Of these, 33 showed no changes, while 10 had at least LA grade B esophagitis (7 after SG, 2 after RYGB, and 1 after OAGB). There were no statistically significant differences in endoscopic signs of GERD after the surgery depending on the type of surgery (*p* = 0.291).

A significantly lower proportion of patients without symptoms of GERD had endoscopic signs of GERD after MBS (12 out of 85, 14.1%) compared to before MBS (39 out of 109, 35.8%) (*p* < 0.001). Among patients with symptoms of GERD before MBS, 30 out of 42 (69.8%) had abnormal endoscopic findings preoperatively, compared to 42 out of 68 (61.8%) postoperatively (*p* = 0.390).

### 3.4. Evolution of Symptoms of GERD Depending on the Surgical Procedure

A total of 78 of 305 patients (12.3%) developed a new onset of symptoms of GERD postoperatively (Table 5). There was no statistical significance (*p* = 0.074). Symptoms of GERD resolved after MBS in 46 of 63 patients (12.5%). Only 17 of 63 patients (27.0%) had persistent symptoms of GERD both before and after MBS. SG was associated with a significantly higher risk of persistence (*p* = 0.005).

## 4. Discussion

Our study describes the outcomes of MBS concerning GERD. To our knowledge, this 10-year follow-up study is among the largest with such an extended follow-up period, within the limited number of studies addressing this topic. Despite the low follow-up rate achieved retrospectively, the strength of the study lies in the detailed description of disease progression, including gastroscopic findings. However, more than 50.0% of patients did not undergo endoscopic evaluation before or after MBS.

In the general population, there are no indications for routine EGD in young adults if they do not have gastrointestinal symptoms. These recommendations are endorsed by both the American College of Gastroenterology and the American Society for Gastrointestinal Endoscopy [18,19]. However, the guidelines differ in the context of MBS. According to the IFSO Position Statement from 2022, EGD should be considered for every patient before MBS, regardless of symptoms, as there is a 25.3% risk of finding an unexpected condition that may lead to a change in the procedure or even serve as a contraindication for MBS [14]. After MBS, EGD is recommended one year and every 2–3 years after SG or OAGB, and when there are upper gastrointestinal symptoms after RYGB. In our study, there were 35.8% abnormal findings in preoperative EGD and 14.2% in postoperative EGD in patients without symptoms of GERD.

Felsenreich et al. reported 10 years reflux outcomes after SG [20]. Even though the presence of HH and preoperative symptoms was a contraindication for SG, postoperative gastroscopies revealed de novo hiatal hernias in 45% of the patients and Barrett’s metaplasia in 15%. A total of 14% of patients were converted to RYGB due to reflux, but in the non-conversion group, 38% of patients suffer from GERD, with some requiring use of PPIs. In our cohort, we observed similar findings. Over 25% of patients who underwent a single procedure reported symptoms of GERD at the 10-year follow-up, and 17.2% of patients who required conversion underwent it due to GERD. The rate of abnormal endoscopic findings was lower than that reported by Felsenreich, but it should be noted that nearly 50% of patients in our cohort did not undergo a postoperative EGD. It is worth noting that we observed a significant decrease in endoscopic signs of GERD in patients without GERD symptoms after MBS compared to before MBS. Kristo et al. demonstrated that GERD affects the majority of patients with obesity, even if they are asymptomatic [21]. This confirms another beneficial effect of bariatric surgery, namely the treatment of GERD.

A Danish nationwide register-based cohort study by Gormsen et al. showed that the risk of initiating and continuing PPI treatment was significantly higher after SG compared to RYGB [22]. After SG, 37% of patients started PPI therapy, compared to 21% after RYGB (HR = 7.06, *p* < 0.001). According to the authors, additional risk factors for PPIs initiation included female sex, older age, smoking, or preoperative use of PPIs. In our study, we did not identify specific predisposing factors, but the Danish cohort included a much larger sample. Another key difference between the procedures in their study was the follow-up duration. Patients who underwent RYGB had a follow-up of up to 11 years, whereas those who underwent SG were followed for only 4 years, which could introduce some bias. The authors reported the continuous increase in PPIs observed after both procedures [22,23]. Other authors have also reported a high incidence of GERD after SG based on pH monitoring in short-term observations [8,9,24]. In a long-term RCT follow-up, Lee et al. found no differences in the incidence of reflux and Barrett’s esophagus between SG and RYGB [25]. Nevertheless, GERD after SG is a significant problem, but other surgical procedures are not without risk either. Kapellas et al., in a meta-analysis, examined the occurrence of GERD after OAGB and RYGB. According to the authors, OAGB was statistically significantly associated with a higher risk of GERD than RYGB (OR = 3.14, *p* < 0.05) [26]. In our 10-year follow-up, we found no statistically significant difference in the occurrence of symptoms of GERD or gastroscopic abnormalities between the surgical procedures performed. However, there is a noticeable trend indicating that SG is associated with a higher rate of GERD compared to RYGB (28.3% vs. 17.5%). Nevertheless, when considering GERD remission after MBS, SG appears to be a procedure associated with a significantly lower likelihood of remission and a significantly higher likelihood of persistent symptoms of GERD compared to RYGB or OAGB. Bevilacqua et al. demonstrated that the risk of new-onset GERD after RYGB is associated with a higher incidence than SG (HR 1.65) [27]. Similarly, in the SM-BOSS study, SG patients experienced significantly more de novo gastroesophageal reflux (GERD) compared to those who underwent RYGB (*p* = 0.02) [28]. In our study, we did not find any significant differences in the incidence of de novo GERD between several types of MBS. The lack of differences may result from the significant numerical advantage of SG over the other procedures. Moreover, our study referred to two time points, which were before the MBS and 10 years after without data for the intermediate years.

An important concept in the case of GERD and MBS, especially SG, is ITM. It can cause symptoms on its own but may also remain asymptomatic [19,29,30]. Crozet et al. demonstrated that 42.4% of patients experienced ITM after SG, and only one-third of them were symptomatic [29]. Moreover, Karila-Cohen et al. reported that even confirmed ITM can be not associated with a positive 24 h pH-monitoring study [30]. To prevent this, Hutopila et al. proposed a novel method involving partial reconstruction of the phreno-esophageal ligament along with hiatal hernia repair [31]. Their results are promising and significantly reduce the risk of ITM compared to hiatal hernia repair alone. In the meta-analysis by Małczak et al., there is also no confirmation of the effectiveness of hiatal hernia repair [32]. However, further research is needed before establishing formal guidelines for clinical practice.

The study has several limitations, most notably the low follow-up rate and its retrospective nature. A low follow-up rate may introduce potential bias in reporting outcomes. It could be assumed that the true prevalence of GERD might be lower than in our analyzed group, as patients without symptoms and complications are more likely to be lost to follow-up. No data were available on patients lost to follow-up to enable comparison of preoperative characteristics. Moreover, our study examines the 10-year period after MBS without a detailed year-by-year analysis. We also lack information on whether symptoms have changed over the years. Additionally, we do not have precise data regarding the percentage of hiatal hernia repairs that were performed. Another limitation of our study is the absence of a standardized, validated questionnaire for symptom assessment. Due to the retrospective design, we were limited to data collected a decade ago, based on clinical interviews recorded uniformly across participating centers. While this ensured consistency, it may limit the precision and comparability of symptom reporting. A major limitation of our study was the incomplete endoscopic data, as more than half of patients did not undergo EGD both before and after MBS. This may lead to an underestimation of the true prevalence of esophageal pathology, especially in asymptomatic patients. The rate of changes observed was based on less than half of the cohort. Similarly, comparisons between pre- and postoperative endoscopic findings should be interpreted with caution, given that only a subset of patients underwent both evaluations. These gaps limit the strength of conclusions regarding the incidence of esophagitis, Barrett’s esophagus, and hiatal hernia, as well as the lack of observed differences between surgical procedures. Future studies should aim to improve adherence to recommended endoscopic surveillance, particularly in light of current guidelines that advocate for routine postoperative EGD after SG and OAGB. Nevertheless, we believe that presenting long-term outcomes in the context of GERD provides important considerations.

## 5. Conclusions

GERD seems to be a common issue after MBS. Over 58% of patients did not undergo preoperative and postoperative EGD. There were 35.8% abnormal findings in preoperative EGD and 14.2% in postoperative EGD in patients without symptoms of GERD. The prevalence at the 10-year follow-up is 25.8%. However, not all patients with symptoms of GERD require chronic use of PPIs. There was no significant difference between the occurrence of postoperative GERD and the type of MBS. SG appears to be associated with a higher risk of persistent GERD and a lower likelihood of GERD remission after MBS. The results should be interpreted with caution due to limitations. Nonetheless, they underscore the importance of monitoring GERD both before and after MBS, as it can develop or persist even in patients without preoperative symptoms.

## Figures and Tables

**Table 1:** Characteristics and outcomes of patients with or without subjective symptoms of gastroesophageal reflux disease before the surgery. (GERD symptoms of gastroesophageal reflux disease, non-GERD no symptoms of gastroesophageal reflux disease, BMI body mass index, IQR interquartile range, SG sleeve gastrectomy, RYGB Roux-en-Y gastric bypass, OAGB one anastomosis gastric bypass, T2D type 2 diabetes, HT hypertension, OBS obstructive sleep apnea, %EWL percentage of excess weight loss, %TWL percentage of total weight loss, PPI proton pump inhibitor).

	GERD *n* = 63	Non-GERD *n* = 305	*p*-Value
*Characteristics*			
Sex, female/male	44/19	209/96	0.837
Age, median (IQR)	42.0 (36.0–50.0)	43.0 (35.0–53.0)	0.939
Preoperative BMI, median (IQR)	40.9 (36.7–45.0)	42.2 (39.3–47.2)	0.013
Type of surgery			
SG, *n* (%)	42 (66.7)	198 (64.9)	0.711
RYGB, *n* (%)	9 (14.3)	56 (18.4)	
OAGB, *n* (%)	12 (19.0)	51 (16.7)	
T2D, *n* (%)	21 (33.3)	92 (30.2)	0.620
HT, *n* (%)	34 (54.0)	160 (52.5)	0.827
OBS, *n* (%)	6 (9.5)	20 (6.6)	0.403
*Outcomes*			
Complications, *n* (%)	4 (6.3)	21 (6.9)	0.878
Postoperative BMI, median (IQR)	33.1 (27.1–35.4)	32.5 (28.4–38.4)	0.100
%EWL, median (IQR)	62.7 (34.0–82.7)	57.1 (29.3–80.1)	0.451
%TWL, median (IQR)	25.0 (12.7–31.9)	22.7 (11.7–32.8)	0.835
Postoperative GERD, *n* (%)	17 (27.0)	78 (25.6)	0.816
PPI postoperative, *n* (%)	13 (20.6)	68 (22.3)	0.772

**Table 2:** Characteristics and outcomes of patients with or without symptoms of GERD after the surgery. (GERD symptoms of gastroesophageal reflux disease, non-GERD no symptoms of gastroesophageal reflux disease, BMI body mass index, IQR interquartile range, SG sleeve gastrectomy, RYGB Roux-en-Y gastric bypass, OAGB one anastomosis gastric bypass, %EWL percentage of excess weight loss, %TWL percentage of total weight loss, PPI proton pump inhibitor).

	GERD *n* = 95	Non-GERD *n* = 273	*p*-Value
*Characteristics*			
Sex, female/male	70/25	183/90	0.228
Age, median (IQR)	42.0 (36.0–50.0)	43.0 (35.0–53.0)	0.733
Preoperative BMI, median (IQR)	42.8 (39.4–45.8)	42.4 (38.6–46.8)	0.983
Type of surgery			
SG, *n* (%)	68 (71.6)	172 (63.0)	0.208
RYGB, *n* (%)	16 (16.8)	49 (17.9)	
OAGB, *n* (%)	11 (11.6)	52 (19.1)	
Preoperative GERD, *n* (%)	17 (17.9)	46 (16.8)	0.816
*Outcomes*			
Complications, *n* (%)	7 (7.4)	18 (6.6)	0.796
Postoperative BMI, median (IQR)	33.1 (29.1–36.9)	32.1 (27.7–38.0)	0.331
%EWL, median (IQR)	58.9 (28.8–75.3)	59.1 (29.4–82.1)	0.465
%TWL, median (IQR)	24.0 (11.6–32.0)	22.4 (12.0–32.6)	0.921
PPI postoperative, *n* (%)	81 (85.3)	0 (0.0)	**<0.001**
Type of surgery among patients with the use of PPI			
SG, *n* (%)	57 (70.4)		0.513
RYGB, *n* (%)	13 (16.0)		
OAGB, *n* (%)	11 (13.6)		

**Table 3:** Univariable logistic regression of the potential factors contributing to symptoms of GERD after surgery. (GERD gastroesophageal reflux disease, OR odds ratio; 95% CI 95% confidence interval, BMI body mass index, SG sleeve gastrectomy, RYGB Roux-en-Y gastric bypass, OAGB one anastomosis gastric bypass, %TWL percentage of total weight loss, T2D type 2 diabetes, HT hypertension).

	OR	95% CI	*p*-Value
Sex, female	1.38	0.82–2.32	0.229
Age	0.96	0.98–1.01	0.597
Preoperative BMI	1.01	0.98–1.04	0.754
Preoperative GERD	1.08	0.58–1.99	0.816
Type of surgery, SG			
RYGB	0.83	0.44–1.55	0.552
OAGB	0.54	0.26–1.09	0.084
T2D	0.81	0.48–1.35	0.413
HT	0.84	0.53–1.34	0.462
%TWL	1.00	0.99–1.01	0.920

**Table 4:** Esophago-gastro-duodenoscopy findings before and after the surgery. (GERD symptoms of gastroesophageal reflux disease, non-GERD no symptoms of gastroesophageal reflux disease, EGD esophago-gastro-duodenoscopy, HH hiatal hernia, LA-A Los Angeles classification grade A esophagitis, LA-B Los Angeles classification grade B esophagitis, LA-C Los Angeles classification grade C esophagitis, LA-D Los Angeles classification grade D esophagitis).

	Preoperative			Postoperative		
	GERD *n* = 63	Non-GERD *n* = 305		GERD *n* = 95	Non-GERD *n* = 273	
*Preoperative EGD*						
No, *n* (%)	**20 (31.8)**	**196 (64.3)**		**52 (54.7)**	**164 (60.1)**	
Yes, *n* (%)	**43 (68.2)**	**109 (35.7)**		**43 (45.3)**	**109 (39.9)**	
No changes	13 (30.2)	70 (64.2)	<0.001	24 (55.8)	59 (54.1)	0.547
HH	20 (46.5)	37 (33.9)		16 (37.2)	41 (37.6)	
LA-A	8 (18.6)	1 (1.0)		3 (7.0)	6 (5.5)	
LA-B	2 (4.7)	1 (1.0)		0 (0.0)	3 (2.8)	
*Postoperative EGD*						
No, *n* (%)	**33 (52.4)**	**182 (59.7)**		**27 (28.4)**	**188 (68.9)**	
Yes, *n* (%)	**30 (47.6)**	**123 (40.3)**		**68 (71.6)**	**85 (31.1)**	
No changes	19 (63.3)	80 (65.0)	0.683	26 (38.2)	73 (85.9)	<0.001
HH	4 (13.3)	7 (5.7)		6 (8.8)	5 (5.9)	
LA-A	1 (3.4)	8 (6.5)		8 (11.8)	1 (1.2)	
LA-B	4 (13.3)	20 (16.3)		20 (29.4)	4 (4.7)	
LA-C/D/Barrett	2 (6.7)	8 (6.5)		8 (11.8)	2 (2.3)	

**Table 5:** Symptoms of GERD depend on the surgical procedure. (GERD gastroesophageal reflux disease, SG sleeve gastrectomy, RYGB Roux-en-Y gastric bypass, OAGB one anastomosis gastric bypass).

Variable, *n*/N * (%)	SG	RYGB	OAGB	*p*-Value
De novo GERD	52/198 (26.3)	16/56 (28.6)	10/51 (19.6)	0.074
Remission of GERD	16/42 (38.1)	9/9 (100)	10/11 (90.9)	0.005
Persistence of GERD	26/42 (61.9)	0/9 (0.0)	1/11 (9.1)	0.005

* N refers to the total for the given procedure.

## Data Availability

The original contributions presented in this study are included in the article. Further inquiries can be directed to the corresponding author(s).
